# Draft Genome Sequences of Two Salmonella Strains Isolated from Wild Animals on the Eastern Shore of Virginia

**DOI:** 10.1128/genomeA.00329-18

**Published:** 2018-05-10

**Authors:** Kevin G. Libuit, Lauren Turner

**Affiliations:** a Division of Consolidated Laboratory Services, Virginia Department of General Services, Richmond, Virginia, USA

## Abstract

Antimicrobial-resistant (AMR) Salmonella infections pose a significant public health threat. Here, we announce two draft genomes of Salmonella strains isolated from wildlife harboring an alarming array of antibiotic resistance genes. Continued investigations of these genomes will provide insight into the possible attribution of AMR Salmonella infection of wild animals.

## GENOME ANNOUNCEMENT

In the United States, antimicrobial-resistant (AMR) nontyphoidal Salmonella infections are considered by the CDC to be a serious threat due to the estimated 100,000 cases of AMR nontyphoidal Salmonella infections occurring annually (1). While extensive investigations of AMR Salmonella infections from clinical settings and livestock exist (2, 3), few studies have focused on wildlife and wild animals, such as deer, geese, ducks, and gulls, and their role in the transmission of AMR Salmonella.

In this report, we announce two draft genome sequences from previously characterized S. enterica subsp. enterica strains isolated from wildlife on the Eastern Shore of Virginia between November 2010 and July 2011 (4).

DNA was extracted from overnight cultures of two Salmonella strains, VA-WGS-00333 and VA-WGS-00353, using the Qiagen DNeasy blood and tissue kit (Valencia, CA, USA). Sequencing libraries for each strain were prepared with DNA using the Illumina Nextera XT kit (San Diego, CA, USA). Sequencing was performed on an Illumina MiSeq using the Illumina 500-cycle kit version 2 according to the manufacturer's instructions.

The serotypes of each strain (VA-WGS-00333: Weslaco; VA-WGS-00353: Singapore) were verified using the *in silico* Salmonella serotype prediction tool SeqSero version 1.0 (5). *De novo* assemblies were generated using SPAdes version 3.9.0 (6), and annotation of the draft genomes was performed using the NCBI Prokaryotic Genome Annotation Pipeline (PGAP) (7).

Quality of the *de novo* assemblies was assessed using Quast version 4.5 (8). The *N*_50_ values of the two assemblies were 278,931 bp and 357,417 bp for VA-WGS-00333 and VA-WGS-00353, respectively. The GC content values (VA-WGS-00333, 52.07%; VA-WGS-00353, 52.18%) and genome lengths (VA-WGS-00333, 4.6 Mb; VA-WGS-00353, 4.7 Mb) were consistent with other reported Salmonella genomes (9, 10).

Annotation of VA-WGS-00333 and VA-WGS-00353 revealed multiple genes that encode proteins associated with antibiotic resistance, including RNase BN, a member of the metallo-beta-lactamase family of proteins (11), 6′-*N*-acetyltransferase, a protein conferring aminoglycoside resistance (12), and various multidrug efflux pumps, such as MdtK, MexE, and EmrA (13–15). The identification of these genes from Salmonella strains isolated directly from wildlife may provide insight into the modes of transmission and outbreak origins of AMR Salmonella strains, particularly when considering the close proximity of migratory birds to agricultural fields. These findings emphasize the importance of continued surveillance and genomic investigations of zoonotic wildlife pathogens and their resistance profiles.

### Accession number(s).

The draft genome sequences for VA-WGS-00333 and VA-WGS-00353 have been deposited in DDBJ/EMBL/GenBank under the accession no. NMOO00000000 and NMON00000000, respectively.
